# Integrated Multi-Omics Reveals DAM-Mediated Phytohormone Regulatory Networks Driving Bud Dormancy in ‘Mixue’ Pears

**DOI:** 10.3390/plants14142172

**Published:** 2025-07-14

**Authors:** Ke-Liang Lyu, Shao-Min Zeng, Xin-Zhong Huang, Cui-Cui Jiang

**Affiliations:** Fruit Research Institute, Fujian Academy of Agricultural Sciences, Fuzhou 350013, China; lvkelianglkl@163.com (K.-L.L.); zengshaomin1@163.com (S.-M.Z.); hxz0117@163.com (X.-Z.H.)

**Keywords:** *Pyrus pyrifolia*, bud dormancy, multi-omics, phytohormone, regulatory network

## Abstract

Pear (*Pyrus pyrifolia*) is an important deciduous fruit tree that requires a specific period of low-temperature accumulation to trigger spring flowering. The warmer winter caused by global warming has led to insufficient winter chilling, disrupting floral initiation and significantly reducing pear yields in Southern China. In this study, we integrated targeted phytohormone metabolomics, full-length transcriptomics, and proteomics to explore the regulatory mechanisms of dormancy in ‘Mixue’, a pear cultivar with an extremely low chilling requirement. Comparative analyses across the multi-omics datasets revealed 30 differentially abundant phytohormone metabolites (DPMs), 2597 differentially expressed proteins (DEPs), and 7722 differentially expressed genes (DEGs). Integrated proteomic and transcriptomic expression clustering analysis identified five members of the dormancy-associated MADS-box (*DAM*) gene family among dormancy-specific differentially expressed proteins (DEPs) and differentially expressed genes (DEGs). Phytohormone correlation analysis and *cis*-regulatory element analysis suggest that *DAM* genes may mediate dormancy progression by responding to abscisic acid (ABA), gibberellin (GA), and salicylic acid (SA). A dormancy-associated transcriptional regulatory network centered on *DAM* genes and phytohormone signaling revealed 35 transcription factors (TFs): 19 TFs appear to directly regulate the expression of *DAM* genes, 18 TFs are transcriptionally regulated by *DAM* genes, and two TFs exhibit bidirectional regulatory interactions with *DAM*. Within this regulatory network, we identified a novel pathway involving *REVEILLE 6* (*RVE6*), *DAM*, and *CONSTANS-LIKE 8* (*COL8*), which might play a critical role in regulating bud dormancy in the ‘Mixue’ low-chilling pear cultivar. Furthermore, lncRNAs *ONT.19912.1* and *ONT.20662.7* exhibit potential *cis*-regulatory interactions with *DAM1/2/3*. This study expands the DAM-mediated transcriptional regulatory network associated with bud dormancy, providing new insights into its molecular regulatory mechanisms in pear and establishing a theoretical framework for future investigations into bud dormancy control.

## 1. Introduction

Bud dormancy is a complex physiological process that is indispensable for the growth, development, and timely flowering of pear buds [1]. During winter, pear buds enter into a state of growth cessation in response to low temperatures. Once a specific period of chilling accumulation—known as the chilling requirement (CR)—is fulfilled, the buds regain the ability to grow and develop [2]. This trait reflects an evolutionary adaptation of higher plants to cold climates through natural selection. Bud dormancy is a critical physiological process that enables plants to survive harsh winter conditions and ensures proper blooming in the subsequent spring.

Endogenous hormones are key regulators of bud dormancy in plants, with abscisic acid (ABA) and gibberellin (GA) serving as primary signals that control the induction and release of dormancy [3,4,5,6]. ABA promotes callose deposition and plasma membrane closure, effectively blocking the transport of growth-promoting substances to the meristem and thereby inducing bud dormancy [3,5,7]. During bud dormancy release, endogenous ABA levels gradually decline in both pear and grape buds [4,8,9]. The exogenous application of ABA delays bud dormancy release in pears, whereas treatment with fluridone, an ABA synthesis inhibitor, accelerates the release of bud dormancy [7]. These findings indicate that ABA plays a central role in maintaining bud dormancy, while GA is also involved in regulating the dormancy release process. After the CR is fulfilled, the expression levels of GA metabolism genes, such as *GA20ox*, *GA3ox*, and *GASA*, increases significantly [5,6]. However, a brief period of low temperatures is still required to initiate dormancy release in buds treated with exogenous GA [5,7,10]. The underlying mechanisms of this process remain unclear. Recent studies have revealed that salicylic acid (SA), jasmonic acid (JA), and brassinosteroid (BR) were also involved in dormancy release. Changes in SA levels and the expression of SA-responsive genes have been observed during bud dormancy release in grapes and litchis [11,12]. Recently, Wang et al. confirmed the involvement of BR and JA in regulating dormancy release in pear buds [13]. These findings suggest that endogenous hormones play a critical role in the regulation of dormancy in plants.

The dormancy-associated MADS-box (*DAM*)/Short Vegetative Phase (*SVP*) gene family functions as a central regulatory hub in the induction and release of plant dormancy. In non-Rosaceae plants, *SVP* genes act as a key transcription factor (TF) involved in ABA signaling, ABA synthesis, GA synthesis, and metabolism, thereby playing a pivotal role in the regulation of endodormancy [14,15]. The *DAM* gene was first identified as part of a tandem repeat of the evergreen mutant of peaches [16]. It is homologous to the *SVP* gene family in *Arabidopsis* and encodes an endodormancy-related MADS-box TF that is specific to *Rosaceae* species [2,17]. The *DAM* gene family is closely linked to hormone signaling and biosynthesis pathways. In the ABA signaling pathway, the expression of C-repeat binding factor (*CBF*) and homeodomain-leucine zipper protein (*HD-ZIP*) family protein PpHB22, both induced by ABA, can independently bind to the promoter region of the *DAM* gene. This binding activates *DAM* transcription and promotes the establishment of endodormancy in pear buds [18,19,20]. Conversely, the ABA-response element-binding TF (*AREB1*) gene has been shown to negatively regulate the expression level of the *DAM1* gene by binding to its promoter region [9]. The *ABRE binding factor* (*ABF*) gene family, which includes *ABF3*, a homolog of *AREB1*, also plays a role in this regulatory network. *ABF3* can bind to the promoter of the *DAM3* gene and enhance its expression. In contrast, *ABF2*, another member of the *ABF* gene family, interacts with the ABF3 protein and interferes with its transcriptional activation function, thereby suppressing the expression level of *DAM3* [7]. The *DAM* gene has been shown to promote the expression of the 9-cis-epoxycarotenoid dioxygenase gene (*NCED*), a key rate-limiting gene in ABA biosynthesis. Elevated expression levels of *NCED*, in turn, contribute to maintaining higher endogenous ABA levels in buds [9]. Additionally, the overexpression of the *DAM3* gene in pear calluses resulted in the downregulation of *Cyclin-D* and *EXPA1*, accompanied by suppressed callus growth, indicating that the *DAM* gene family may also play a role in regulating cell division [7].

Southern China is a warm, early-ripening pear producing region. Due to its relatively mild winters, the duration of chilling accumulation is short. As a result, farmers in this region prefer to cultivate pear varieties with low CR to ensure the proper induction and release of bud dormancy during winter. However, the increasing frequency of warm winters caused by global warming has often led to insufficient chilling accumulation, posing a challenge to dormancy release and subsequent bud development [21].

‘Mixue’ is a pear cultivar with extremely low CR, capable of inducing and releasing bud dormancy even under warmer winter conditions. This trait is particularly valuable for pear cultivation and production in Southern China, where mild winters are common. In this study, the full-length transcriptomes of ’Mixue’ pear buds at four developmental stages were used to improve the annotation of the pear reference genome. Integrated metabolomic, transcriptomic, and proteomic analyses revealed that, in addition to ABA and GA, JA, and SA, strigolactone (SL) also participate in regulating bud dormancy in this pear with a low CR. Based on these findings, we constructed a hormone-related expression regulatory network to identify key genes responsive to hormonal signals during dormancy regulation. This study provides a theoretical foundation for elucidating the molecular mechanisms underlying bud dormancy in low CR pear cultivars and offers valuable insights for improving pear yield and fruit quality.

## 2. Results

### 2.1. Paraffin Section Analysis of Pear Buds from Different Developmental Stages

**Figure 1:**
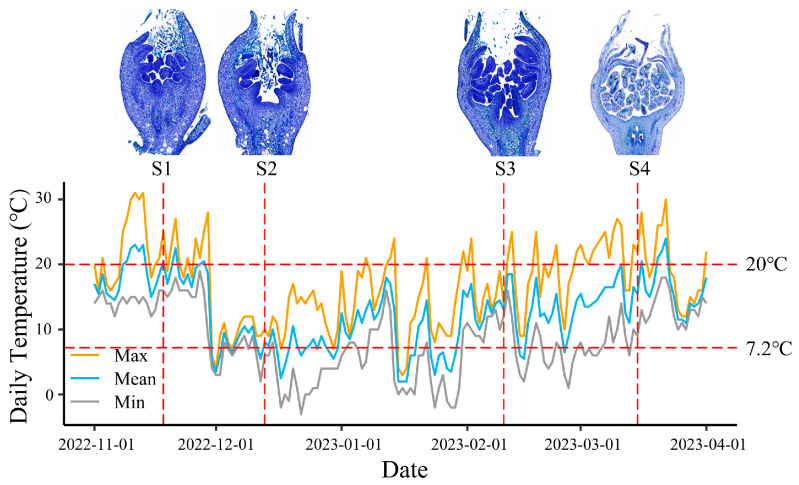
The paraffin sections of buds at four different periods, and the mean daily temperatures of Qingliu from November 2022 to March 2023.

To investigate the regulatory mechanisms of endodormancy, bud sampling was carried out based on daily mean temperatures in Qingliu from November 2022 to March 2023. Samples were collected at four distinct developmental stages, ranging from before the onset of chilling accumulation (defined as three consecutive days with temperatures below 7.2 °C) to just before bud break. These stages were designated as S1 (pre-chilling accumulation), S2 (early chilling accumulation), S3 (late chilling accumulation), and S4 (pre-bud break) (Figure 1). To clarify the developmental status of ‘Mixue’ pear buds at each stage, paraffin sectioning was performed (Figure 1). The results showed that at S1, stamens had differentiated from the floral primordium. At S2, pistil differentiation was evident. By S3, the initiation of carpel closure was observed, indicating the onset of pistil carpel fusion. At the final stage, S4, the carpels were fully closed and the ovary cavity (ventricle) was clearly visible.

### 2.2. Metabolomic Analysis of Endogenous Phytohormones in Pear Buds

Endogenous phytohormones play pivotal roles in regulating plant growth and development, acting as both key regulatory factors and biomarkers during the stages of bud dormancy. To explore the dynamic changes in phytohormone levels in the buds of the low CR pear cultivar ‘Mixue’, a targeted metabolomic analysis was performed using liquid chromatography–tandem mass spectrometry (LC-MS/MS) on four developmental stages of buds before bud break. A total of 59 endogenous phytohormone metabolites were identified in this metabolomic dataset, with 30 metabolites characterized as the differentially abundant phytohormone metabolites (DPMs). These DPMs include two ABA-related metabolites, ten auxin-related metabolites, four cytokinin (CK)-related metabolites, one ethylene (ETH)-related metabolite, five GA-related metabolites, five jasmonic acid (JA)-related metabolites, two SA-related metabolites, and one strigolactone (SL)-related metabolite (Supplementary Table S1).

**Figure 2:**
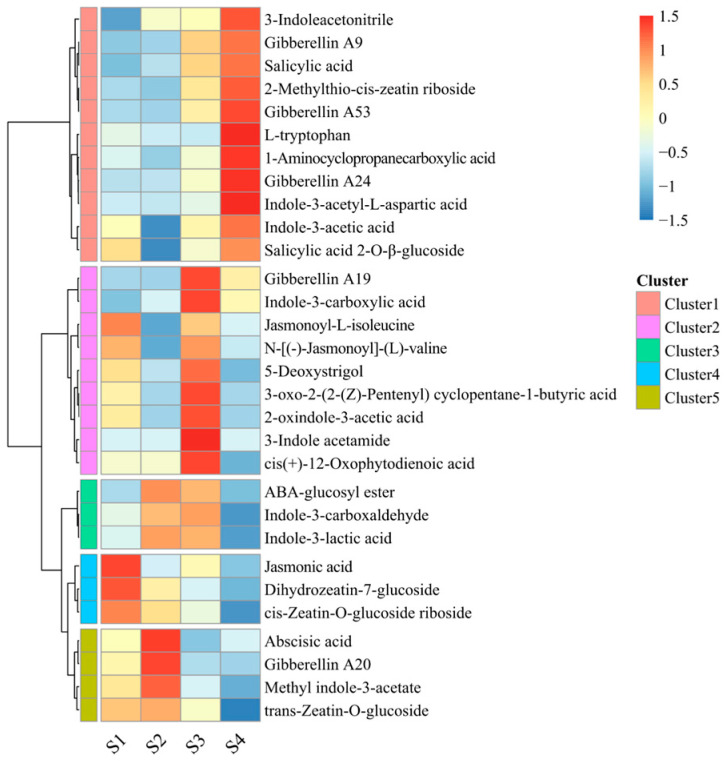
The heatmap of differential endogenous hormone metabolites in ‘Mixue’ buds during four developmental stages. Different colors indicate the expression levels of DPMs, from blue (low) to red (high), as indicated in the top-right panel.

Based on the accumulation profiles of these phytohormone metabolites across distinct phases before and after bud dormancy, abundance-based clustering analysis classified them into five groups (Figure 2). Metabolites exhibiting the highest accumulation levels at the S4 stage were classified into Cluster 1, including four auxin-related metabolites, one CK-related metabolite, one ETH-related metabolite, three GA-related metabolites, and two SA-related metabolites. Metabolites grouped into Cluster 2 (including three auxin-related metabolites, one GA-related metabolite, four JA-related metabolites, and one SL-related metabolite), exhibited a markedly elevating accumulation at the S3 stage, when compared to the other three stages. The metabolites of Cluster 3, comprising one ABA-related metabolite and two auxin-related metabolites, were predominantly accumulated during the S2 and S3 stages. The metabolites in Cluster 4, including one CK-related metabolite and one JA-related metabolite, have the highest accumulation levels at the S1 stage. Metabolites showing peak accumulation levels at the S2 stage were classified into Cluster 5, including ABA, one auxin-related metabolite, one CK-related metabolite, and one GA-related metabolite (Figure 2). Metabolites in Clusters 1, 2, and 5 displayed clear stage-specific patterns related to bud dormancy. These clusters encompassed the majority of metabolites associated with ABA, GA, JA, SA, and SL. ABA-related metabolites, primarily grouped in Cluster 4, reached their highest levels prior to dormancy onset and declined sharply following dormancy release. In contrast, GA-, JA-, SA-, and SL-related metabolites were mainly enriched in Clusters 1 and 2, showing low abundance before dormancy and increasing significantly after dormancy release. This dormancy-specific accumulation pattern suggests that these phytohormones may play critical roles in regulating bud dormancy in pear (Figure 2).

### 2.3. Improvement of Reference Genome Annotation

**Figure 3:**
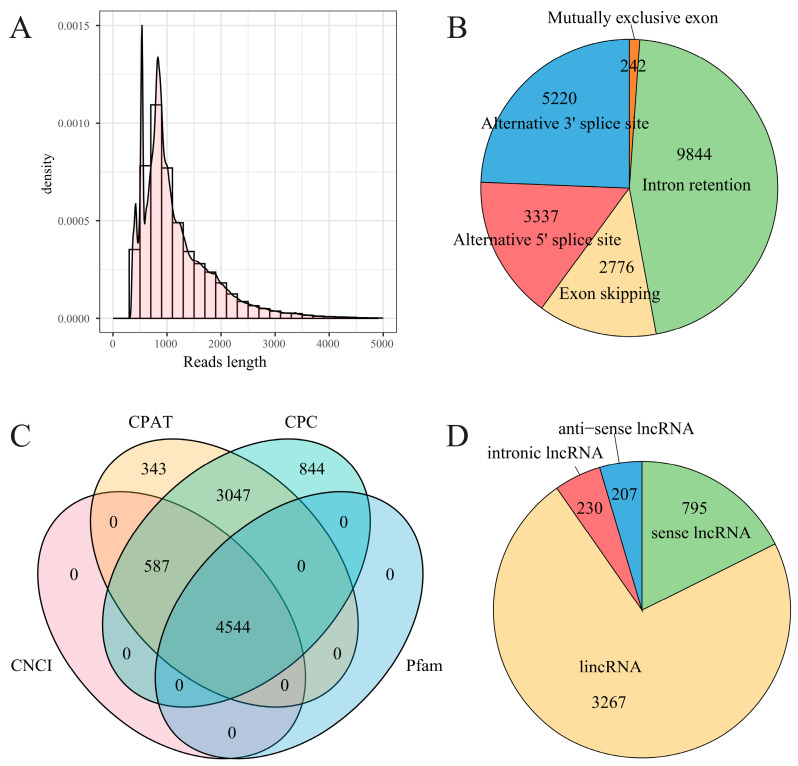
A summary of the full-length transcriptome dataset. (**A**) The histogram of clean read length distribution from the full-length transcriptome sequencing dataset; (**B**) the pie plot of AS event types annotated in the full-length transcriptome dataset; (**C**) the Venn diagram illustrating the overlap between the lncRNAs identified by four different software. The red, yellow, green, and blue ellipses represent the lncRNAs predicted by the Coding–Non-Coding Index (CNCI), Coding Potential Calculator (CPC), Coding Potential Assessment Tool (CPAT), and Pfam algorithms, respectively; (**D**) The pie plot illustrates the distribution of different types of lncRNAs. The four colors (red, yellow, green, and blue) represent the four main types of lncRNA, namely intergenic lncRNA (lincRNA), intronic lncRNA, sense lncRNA, and anti-sense lncRNA, respectively.

The reference genome serves as the foundation for genomic and transcriptomic analyses, and the quality of genome annotation significantly influences the accuracy and reliability of genomic and transcriptomic data interpretation. The annotation of the pear reference genome Cuiguan V1.0 shows deficiencies in untranslated regions (UTRs) and alternative splicing (AS) information [17]. To improve the annotation of UTRs and AS events, full-length transcriptome sequencing across four developmental stages before and after bud dormancy in pears was performed using the ONT third-generation sequencing platform MinION. After filtering out rRNA and low-quality reads, a total of 71.82 Mb clean reads (84.73 Gb bases) were obtained, with full-length sequences accounting for 92% of each sample dataset (Supplementary Table S2). The clean reads in the dataset had an average length of 1179.90 bp, with the longest read spanning 270,346 bp and the shortest read 350 bp (Figure 3A). These results demonstrate the high completeness of the full-length transcriptome dataset, confirming its suitability for downstream analyses. Alignment of deduplicated reads to the reference genome identified 4602 novel genes and 24,500 novel transcripts (Supplementary Tables S2–S4). Comparative analysis with existing genome annotations refined the boundaries of 17,091 genes, including 14,112 transcription start sites and 14,917 transcription termination sites (Supplementary Table S5). Additionally, 21,419 AS events were identified across 8995 genes, with the following distribution: 9844 intron retention, 12.96% exon skipping, 242 mutually exclusive exons, 3337 alternative 5′ splice sites, and 5220 alternative 3′ splice sites (Figure 3B, Supplementary Table S6). The predominant AS types during dormancy progression in ‘Mixue’ pear were intron retention and alternative 3′ splice sites, together accounting for 69.34% of all AS events.

The novel transcripts were subjected to multiple analytical pipelines, including the Coding–Non-Coding Index (CNCI), the Coding Potential Calculator (CPC), the Coding Potential Assessment Tool (CPAT), and Pfam protein structure domain identification [22,23,24,25], to identify long non-coding RNAs (lncRNAs) (Figure 3C; Supplementary Table S7). A total of 4544 lncRNAs were identified, including 3277 intergenic lncRNAs (lincRNA), 240 intronic lncRNAs, 795 sense lncRNAs, and 232 antisense lncRNAs (Figure 3D; Supplementary Table S8). These identified lncRNAs provide a valuable resource for further investigation into their roles in the regulation of bud dormancy in low-CR pear cultivars. In addition, we predicted the regulatory potential of these lncRNAs and identified a total of 5974 lncRNAs with putative regulatory functions (Supplementary Table S9).

### 2.4. Differenially Expressed Genes (DEGs) and Differentially Expressed Proteins (DEPs)

**Figure 4:**
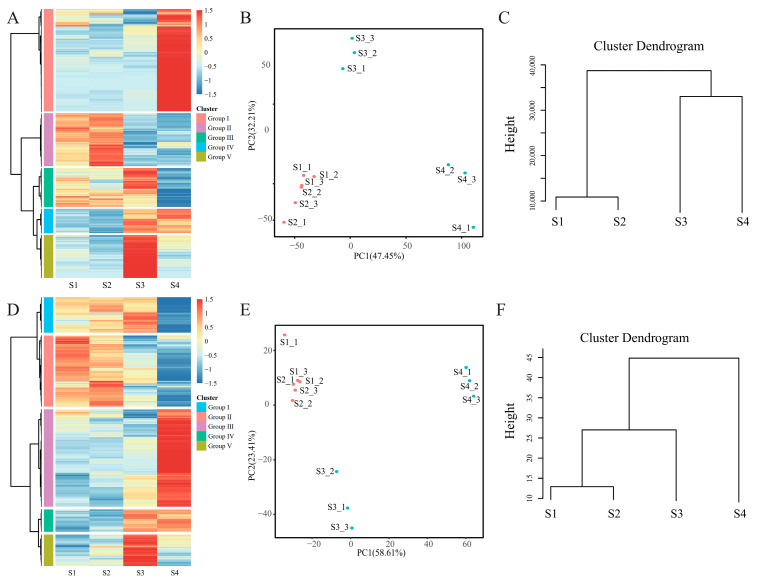
Transcriptome and proteome data analysis at four developmental stages of ‘Mixue’ buds. (**A**,**D**) Represent the expression heatmap of DEGs and DEPs, respectively. The color spectrum is used to denote the Z-score of the expression level, with red representing the highest and blue the lowest. (**B**,**E**) Are dot plots illustrating PCA results; the former represents PCA of DEGs, and the latter represents PCA of DEPs. The X-axis and Y-axis of these dot plots represent PC1 and PC2, respectively. (**C**,**F**) Are the cluster dendrograms of DEGs and DEPs.

To investigate transcriptional changes across developmental stages and identify key genes involved in bud dormancy regulated by endogenous hormones, a total of 7722 DEGs were identified in ‘Mixue’ pear buds across four developmental stages. Based on their expression patterns during bud development, these DEGs were grouped into five distinct clusters (Figure 4A; Supplementary Table S10). Group I (39.43%, 3045 of 7722) genes are specifically expressed at the S4 stage. Group II (20.19%, 1559 of 7722) genes are predominantly expressed before bud dormancy. Group III (14.79%, 1142 of 7722) genes are expressed at all stages except the S4 stage. Group IV (9.22%, 712 of 7722) genes are specifically expressed after dormancy release. The expression level of Group V (16.37%, 1264 of 7722) genes increased significantly after dormancy release, and then decreased rapidly. Group II, Group IV, and Group V exhibited distinct stage-specific expression patterns before dormancy and after dormancy.

To investigate the differential protein expression at various developmental stages of ‘Mixue’ buds, the proteomic dataset was mapped to the NR database, and a total of 7492 proteins were identified, of which 2597 proteins were identified as DEPs at four developmental stages (Supplementary Tables S11 and S12). Based on the expression patterns, these DEPs could be divided into five clusters (Figure 4D). Group I (27.57%, 716 of 2597) is expressed in all samples except S4. Group II (37.78%, 981 of 2597) is specifically expressed before bud dormancy. Group III (8.70%, 226 of 2597) is specifically expressed at the S4 stage. Group IV (13.79%, 358 of 2597) is specifically expressed after buds break dormancy. Group V (12.17%, 316 of 2597) increased significantly after breaking dormancy, followed by a rapid decline. Overall, both the proteome and transcriptome exhibited clear developmental specificity during bud differentiation in ‘Mixue’ pears (Figure 4B,C,E,F).

To assess proteome–transcriptome concordance, the identified proteins were aligned to the reference genome to map corresponding transcripts. Correlation between these protein–transcript pairs was calculated and visualized (Supplementary Figure S1). The results show a strong correlation (PCC ≥ 0.75) for most proteins, indicating good concordance between the proteomic and transcriptomic datasets.

### 2.5. Identification of Key Genes Involved in the Regulation of Dormancy

**Figure 5:**
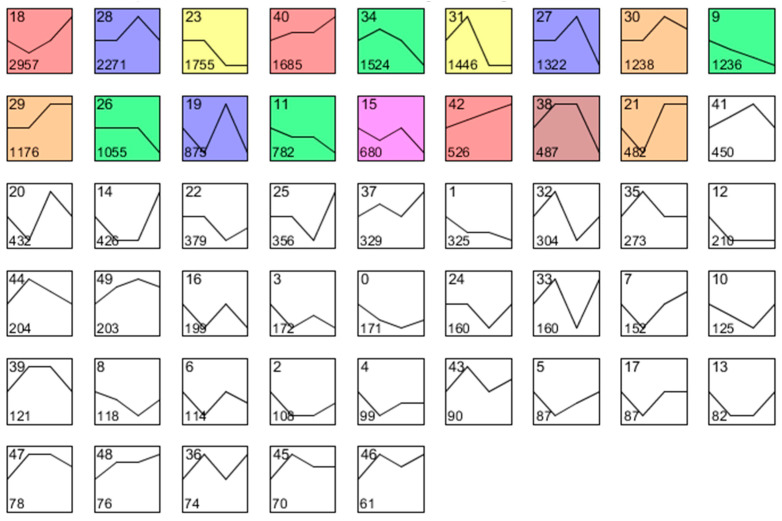
Module profiles of the DEGs at four developmental stages of ‘Mixue’ buds. Each rectangle represents a model profile. The lines in the rectangles represent the expression patterns of DEGs at four developmental stages. The numerals situated in the upper left and lower left of the rectangle represent the model profile number and the number of genes, respectively. The color of the rectangle represents a different expression cluster.

To identify key genes involved in regulating bud dormancy, we further analyzed the expression patterns of all DEGs across four developmental stages of pear buds (Figure 5). A total of 17 statistically significant model profiles was identified by using Short Time-series Expression Miner (STEM), and were grouped into seven clusters (with each colored rectangle representing a different cluster). Among these profiles, the blue cluster (including profile 19, profile 27, and profile 28), the yellow cluster (including profile 23 and profile 31), and the tan cluster (including profile 21, profile 29, and profile 30) exhibited distinct stage-specific expression patterns associated with the bud dormancy process (Figure 5). The expression levels in the blue and tan clusters exhibited a notable increase after dormancy release, whereas the expression levels of genes in the yellow cluster decreased significantly after dormancy release. Through STEM analysis and gene functional annotation, combined with a clustering analysis of DEGs and DEPs, all family members of the *DAM* gene family related to hormone-regulated bud dormancy were identified within the yellow cluster. Specifically, *DAM1*, *DAM2*, *DAM3*, and *DAM4-2* were found in profile 23 and *DAM4-1* was found in profile 31. All these *DAM* genes were also present in Group II of the DEGs cluster, and clustered within Group II of both DEGs and DEPs, exhibiting dormancy-specific expression patterns (Figure 4).

**Figure 6:**
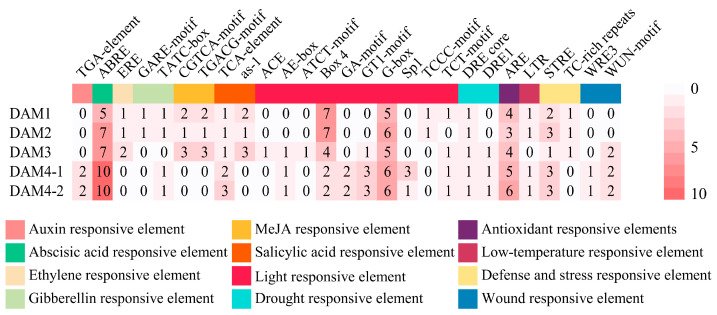
The responsive elements in the promoters of the *DAM* gene family. The color red on the heatmap denotes the quantity of responsive elements; white represents a value of 0 and red indicates a value of 10. The colored rectangle displayed above the heatmap represents the response function of the elements.

To gain further insight into the relationship between *DAM* genes and hormones, Pearson correlation coefficients (PCCs) were calculated between the expression levels of the *DAM* gene family and hormone accumulation. The results show a strong positive correlation between *DAM* gene expression levels and most of the DPMs (Table 1). It is noteworthy that all GAs, except for GA20, exhibited a strong negative correlation with *DAM* gene expression levels (PCC < −0.79), whereas GA20 showed a strong positive correlation (PCC > 0.75). Additionally, methylindole-3-acetate demonstrated a strong negative correlation with *DAM4-1*, while 2-methylthio-cis-zeatin riboside exhibited a significant negative association with *DAM4-2*. Among these GA-related metabolites, GA9 is significantly negatively correlated with *DAM1/4-1*. Furthermore, a strong negative relationship exists between methylindole-3-acetate and *DAM4-1*, as well as between 2-methylthio-cis-zeatin riboside and *DAM4-2*. Similarly, there is a significant negative correlation between SA and *DAM1/2*. These metabolites are likely directly involved in regulating the transcriptional activity of the *DAM* gene family. To investigate this further, promoter sequences of the *DAM* gene family were extracted from the reference genome (Figure 6, Supplementary Table S13), aiming to characterize the response features of the *DAM* gene. The *DAM* gene family responds to a variety of stimuli, including different hormones. This finding is consistent with the results of Pearson correlation analysis mentioned above (Table 1). All promoters of the *DAM* gene family contain numerous light-responsive and ABA-responsive elements, along with a limited number of SA-responsive elements. Furthermore, the distribution of auxin-, MeJA-, and ethylene-responsive elements in the promoters of the *DAM* gene family is chromosome-specific (Figure 6; Supplementary Table S14). MeJA- and ethylene-responsive elements were identified exclusively within the promoters of the *DAM1*, *DAM2*, and *DAM3*, which are located at the same locus on chromosome 8. Auxin-responsive elements are present only in the promoters of *DAM4-1* and *DAM4-2*, located at different loci in chromosome 15. These results demonstrate that the DAM gene family responds to auxin, MeJA, and ethylene in a gene cluster-specific manner, suggesting that different DAM clusters play distinct roles in regulating bud dormancy under hormonal control.

**Figure 7:**
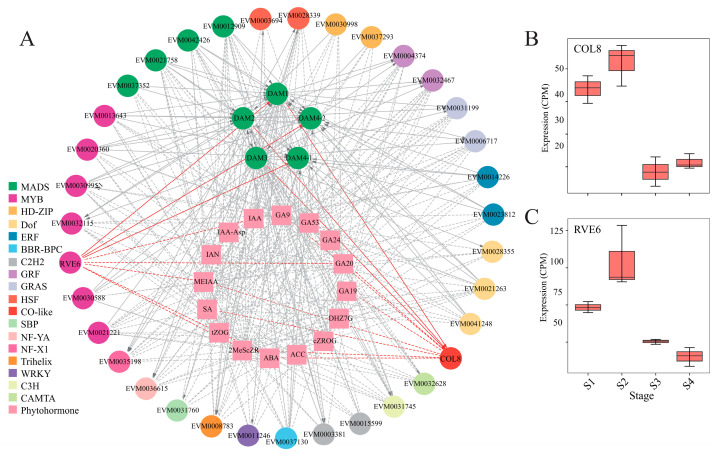
The co-expression regulatory network of the *DAM* gene family. (**A**) The differential gene co-expression regulatory network based on the *DAM* gene family. The different colors represent different TF families. The rectangle represents phytohormones; the ellipse represents TFs. All lines represent pairwise correlations with |Pearson correlation coefficient (PCC)| > 0.9, where solid lines indicate statistically significant relationships (*p* < 0.05), and dashed +lines denote non-significant correlations (*p* ≥ 0.05). The arrow in the figure represents a regulatory relationship. The red line represents the regulatory network of *RVE6* and *COL8*; (**B**,**C**) are the box plots of the expression levels of the *RVE6* and *COL8* genes, respectively.

To identify the function genes and regulatory factors involved in bud dormancy, the regulatory effects of the *DAM* gene family on other genes during the four developmental stages of ‘Mixue’ pear buds were analyzed. Gene expression data from Group II and predicted TF regulation were used to build the expression regulation network for the *DAM* gene family (Figure 7A, Supplementary Table S15). We identified 35 TFs and 169 function genes in this expression regulatory network. Among them, 18 TFs (including 12 *MADS*, 3 *MYBs*, one *Dof*, and one *HD-ZIP*) were predicted to regulate the expression of *DAM* genes. Through gene functional annotation, we found that all of these TFs are associated with flowering. The expression levels of 16 TFs (including one *C3H*, one *CAMTA*, one *CO-like*, one *Dof*, two *GRASs*, two *GRFs*, two *HSFs*, one *MYB*, one *NF-X1*, one *NF-YA*, one *SBP*, one Trihelix, and one *WRKY*) were regulated by *DAM* genes. Additionally, two TFs (one *C2H2* and one *ERF*) exhibited mutual regulatory relationships with the *DAM* genes. These TFs were suggested to play important roles in regulating bud dormancy in ‘Mixue’ pears. Furthermore, a highly expressed circadian clock gene, *RVE6,* was found to regulate the expression levels of *DAM1*, *DAM4-1*, and *DAM4-2*, while another highly expressed circadian clock gene, *COL8*, was regulated by *DAM1*, *DAM2*, and *DAM3* genes (Figure 7). Both RVE6 and COL8 were highly expressed at the S1 and S2 stages (Figure 7B,C).

In this study, a total of 20 lncRNAs with the potential to regulate *DAM* genes were identified based on lncRNA regulation prediction results (Supplementary Table S9). To further analyze their regulatory potential, we performed an expression level correlation analysis on these 20 lncRNAs and the *DAM* gene family. The results showed that the expression levels of *ONT.19912.1* and *ONT.20662.7* were significantly positively correlated with the *DAM* gene family (Supplementary Table S16). Sequence alignment revealed that *ONT.20662.7* is homologous to the *DAM* genes (Supplementary Figure S2).

## 3. Discussion

Bud dormancy is a physiological state in which plant buds temporarily cease growth and development, resuming activity only after a specific CR has been fulfilled [26]. In Southern China, the duration of the chilling accumulation period is relatively short, and with the progression of global warming, insufficient chilling has become increasingly common in pear trees. This deficiency leads to reduced flowering and fruit yield, resulting in significant economic losses for farmers. The pear cultivar ‘Mixue’ exhibits a low CR, allowing for normal flowering even under climatic conditions with extremely short chilling accumulation periods. This trait holds substantial importance for the pear industry in Southern China. However, to date, no studies have investigated the mechanisms underlying dormancy release in ‘Mixue’ pears using integrated transcriptomic, proteomic, and metabolomic approaches. In this study, we employed multi-omics analyses to examine the differentiation of ‘Mixue’ pear buds at four developmental stages, spanning from the period prior to chilling accumulation (defined as three consecutive days with average temperatures below 7.2 °C) to the pre-bud break stage (Figure 1). The objective was to detect and characterize the associated metabolic, proteomic, and transcriptomic profiles, thereby providing a valuable resource for elucidating the transcriptional regulatory mechanisms underlying bud dormancy in low-CR pear cultivars.

### 3.1. Improved Transcription Annotation of the Pear Reference Genome Is Essential for Analyzing the Mechanisms That Regulate Dormancy

The accurate annotation of untranslated regions (UTRs) and AS isoforms in reference genomes is essential for deciphering gene expression regulatory mechanisms and advancing molecular breeding. The current annotation of the pear reference genome Cuiguan V1.0 exhibits deficiencies in both untranslated regions (UTRs) and AS information [17]. UTRs play crucial roles in regulating mRNA stability, translational efficiency, and responses to environmental stress. Studies have demonstrated that the length and sequence of 3′UTRs can significantly influence mRNA stability. Longer 3′UTRs may activate nonsense-mediated mRNA decay (NMD), a surveillance mechanism that eliminates aberrant transcripts containing premature termination codons (PTCs), thereby preventing the accumulation of truncated and potentially harmful proteins [27]. In *Arabidopsis*, UTR polymorphisms are directly associated with temperature adaptability and root hydraulic conductivity, where specific UTR variants enhance drought tolerance by modulating mRNA stability, translational efficiency, and stress-responsive protein synthesis [27]. In this study, full-length transcriptome data from four bud developmental stages were used to improve the reference genome annotation. A total of 17,091 gene boundaries were redefined, with 14,112 5′UTRs and 14,917 3′UTRs extended, thereby establishing a robust data foundation for investigating UTR-mediated transcriptional regulation and stress-responsive mechanisms (Figure 3, Supplementary Table S5). AS generates diverse mRNA transcripts through exon skipping, intron retention, or alternative donor/acceptor site usage, significantly expanding proteome diversity. This mechanism serves as a key adaptive strategy for plants, allowing them to adjust protein isoforms in response to changing conditions. In *Arabidopsis*, heat shock proteins (*HSPs*) undergo AS to produce membrane-localized or cytosolic isoforms, which are functionally specialized for thermotolerance and basal metabolic regulation, respectively [27]. REVEILLE 2 (*RVE2*) achieves rapid cold signaling responses (<20 min) by dynamically modulating AS ratios and isoform-specific expression levels, thereby enabling swift transcriptional reprogramming under low-temperature stress [28]. In this study, 21,419 AS events across 8995 genes were annotated (Supplementary Table S6). The refined AS annotation in the reference genome greatly enhances functional genomics research and provides a robust data foundation for high-precision, high-throughput investigations into gene regulatory networks and phenotypic associations.

### 3.2. A Number of Phytohormones Are Involved in the Regulation of Bud Dormancy in Pear Cultivars with Low CR

Phytohormones act as key regulatory factors in various plant physiological processes and play a pivotal role in dormancy regulation [13]. In the targeted phytohormone metabolomics dataset spanning four dormancy and developmental stages of pear buds, 30 DPMs were identified (Figure 2). Among them, ABA and GA are the most extensive phytohormones in dormancy research. ABA is a critical factor in the induction and maintenance of bud dormancy, the exogenous application of ABA delays dormancy release in pear buds, while treatment with fluridone (an ABA biosynthesis inhibitor) accelerates dormancy release [7]. GA content is closely correlated with dormancy release. After exposure to low-temperature treatment, the expression levels of GA biosynthesis-related genes (*GA20ox*, *GA3ox*, and *GASA*) in dormant buds were significantly upregulated, accompanied by a marked increase in endogenous GA content [5,6]. In this study, the accumulation patterns of ABA and GA were consistent with previous findings [5,6,7]. During dormancy progression (Figure 1; stages S1 and S2), ABA levels continuously increased and reached a peak. Most GA-related DPMs exhibited sustained accumulation throughout dormancy progression, with peaking levels occurring after bud dormancy release (stages S3 or S4). Notably, GA20 exhibited an accumulation pattern associated with dormancy progression that was distinct from other GA-related DPMs. Its levels continuously increased and peaked during dormancy progression (stages S1 and S2), but dropped to extremely low levels after dormancy release (stages S3 and S4). This suggests that GA20 biosynthesis is regulated by a mechanism distinct from that of other GA-related DPMs during dormancy progression. GA20 content was also found to increase significantly in beechnut seeds following dormancy-breaking treatment [29]. These findings indicate a unique functional role of GA20 in regulating bud dormancy release in ‘Mixue’ pears.

SA and JA also play pivotal regulatory roles in bud dormancy progression. Analysis of phytohormone-targeted DPM accumulation patterns (Figure 3) revealed that SA levels continuously increased during dormancy progression, peaking just before the bud break stage (stage S4). Its accumulation levels were significantly positively correlated with the expression of dormancy-associated core genes *DAM1* and *DAM2* (Table 1). Promoter analysis identified multiple SA-responsive *cis*-acting elements in the *DAM1* and *DAM2* promoters (Figure 6), collectively suggesting that SA likely regulates dormancy release by modulating the expression levels of these genes. Transcriptomic studies of litchi bud dormancy also observed significant enrichment of the SA signaling pathway during dormancy release [11]. Additionally, SA has received considerable attention for its role in plant cold resistance and cold signal transduction [30]. These findings suggest a potential link between SA and chilling accumulation during dormancy progression in ‘Mixue’ pears. Notably, JA accumulation levels in this study showed a weak correlation with the expression levels of core dormancy-associated *DAM* genes. However, multiple JA-responsive *cis*-regulatory elements were identified in the promoters of *DAM1* and *DAM3*. Recent studies suggest that JA accumulation in pear buds interacts with the BR-signaling pathway during the regulation of dormancy release. Low JA concentrations promote dormancy release, whereas high JA concentrations suppress it, indicating a complex concentration-dependent regulatory mechanism [13]. This mechanistic complexity likely contributes to the low correlation between JA accumulation levels and *DAM* expression observed in the current study.

Promoter analysis of the dormancy-associated core gene family *DAM* revealed that pear dormancy progression is regulated by multiple phytohormones (Figure 6). The promoters of the dormancy-associated *DAM* gene family contain multiple ABA-responsive and antioxidant response elements, consistent with previous studies showing that ABA accumulation levels and elevated peroxide metabolism during dormancy progression reciprocally modulate *DAM* gene expression [31]. Furthermore, the promoters of multiple *DAM* family genes contain *cis*-regulatory elements responsive to auxin, ETH, GA, MeJA, and SA (Figure 6), indicating that *DAM* genes are regulated by multiple phytohormones. As core regulators of dormancy, the expression levels of *DAM* genes are critical factors in pear bud dormancy regulation. These findings collectively suggest that dormancy induction and release in ‘Mixue’ pears result from multifactorial hormonal regulation. Interestingly, *cis*-regulatory elements responsive to auxin, MeJA, and ETH exhibit locus-specific distribution patterns within the promoters of the *DAM* gene family (Figure 6). MeJA- and ETH-responsive elements are exclusively present in the promoters of *DAM1/2/3*, which are co-localized within the same genome locus. In contrast, auxin-responsive *cis*-regulatory elements are found within the promoters of *DAM4-1/4-2*, which reside in a distinct genomic locus. The locus-specific distribution of these *cis*-regulatory elements suggests that, during the evolution of the pear *DAM* gene family, responsiveness to particular phytohormones became genetically fixed at particular loci, potentially conferring distinct regulatory functions in the control of bud dormancy in the ‘Mixue’ pear cultivar.

### 3.3. The Identification of Key Genes Involved in Dormancy Regulation During the Dormancy Progression of Low-Chilling-Requirement Pear Buds

The *DAM* gene family, a Rosaceae-specific group of dormancy-associated MADS-box transcription factors, plays a pivotal regulatory role in bud dormancy across Rosaceae species [32]. The reference genome of ‘Cuiguan’ pears contains five *DAM* gene family members, organized in tandem arrays at two loci: chromosome 8 (loci with *DAM1*/2/3) and chromosome 15 (loci with *DAM4-1*/*4-2*) [17]. Previous studies have demonstrated that the *DAM* gene family plays critical roles in both the induction and release of plant dormancy, serving as core regulators of dormancy control [7,13,17,18,19,20,32]. In this study, five *DAM* gene family members were identified as co-enriched in two dormancy-specific expression clusters (the Group II cluster of DEGs and the yellow profile from time series analysis) through expression pattern clustering and temporal expression profiling (Figure 4A and Figure 5). During dormancy progression (stages S1 and S2), their expression levels gradually increased and peaked, while after dormancy release (stages S3 and S4), their expression declined, demonstrating a dormancy-specific expression pattern. These results suggest that the *DAM* gene family plays a regulatory role in dormancy progression in ‘Mixue’ pears.

**Figure 8:**
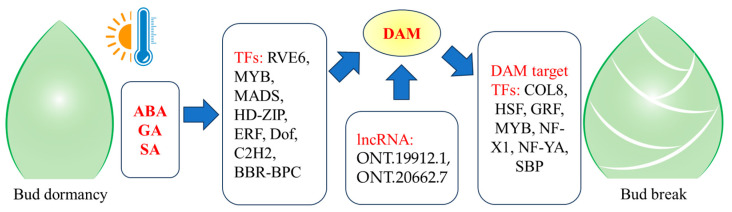
The presumed model of DAM-mediated dormancy regulation during the bud dormancy progression of ‘Mixue’ pears.

A regulatory network centered on the *DAM* gene family and phytohormone signaling was constructed based on transcriptional regulation predictions and gene co-expression analysis, enabling the identification of key genes involved in dormancy regulation and the elucidation of mechanisms driving dormancy progression. A total of 35 TFs with potential regulatory relationships to the *DAM* gene family were identified (Figure 7). Among these TFs, 19 TFs were predicted to directly bind to *DAM* promoters to regulate their expression, while 18 TFs were transcriptionally regulated by *DAM* genes. These TFs exhibit strong correlations with the accumulation levels of 16 differentially produced phytohormone metabolites, suggesting their potential role in mediating hormone signaling to regulate pear bud dormancy. Based on the expression regulatory network, we constructed a diagram illustrating the DAM-mediated regulatory mechanism for dormancy control (Figure 7 and Figure 8, Supplementary Table S9). A series of novel regulatory relationships were uncovered within these regulation mechanisms, revealing previously unrecognized pathways involved in pear dormancy progression. The MYB transcription factor *RVE6* regulates the expression levels of *DAM1*, *DAM4-1*, and *DAM4-2* by binding to their promoter regions. Concurrently, the *DAM1*, *DAM2*, and *DAM3* genes bind to the promoter of CONSTANS-like 8 (*COL8*) and activate its transcription. Together, these interactions form the RVE-DAM-COL regulatory pathway [33,34]. Studies have shown that *RVE* transcription factors also play critical roles in temperature responsiveness and plant dormancy regulation. In *Arabidopsis*, cold stress rapidly induces AS of *RVE2* (within <20 min), increasing the proportion of functional transcripts and enhancing *RVE2* gene expression levels, thereby enabling swift adaptation to low-temperature signals [28]. The expression levels of *RVE1* and *RVE2* are suppressed by red light signaling, which alleviates their transcriptional repression of *GA3ox2*, leading to enhanced GA biosynthesis and GA-dependent seed dormancy release [35]. COL proteins function as transcriptional repressors of the flowering genes *FT* and *SOC1*, whereas *FT* acts as a key growth signal promoting bud dormancy release. These findings suggest that the RVE-DAM-COL regulatory pathway represents a distinct dormancy-regulating mechanism, playing a central role in orchestrating dormancy progression in low-CR pear cultivars such as ‘Mixue’.

### 3.4. Identification of lncRNAs Provides Data Support for Screening Potential Dormancy-Related Competing Endogenous RNAs (ceRNAs)

The lncRNAs play crucial regulatory roles in plant flowering and dormancy processes by modulating epigenetic modifications, chromatin remodeling, and transcriptional/post-transcriptional regulation [2,36,37]. In *Arabidopsis*, *COOLAIR* (cold-induced antisense intragenic RNA) and *COLDAIR* (cold-assisted intronic non-coding RNA) lncRNAs silence the Flowering Locus C (*FLC*), a key flowering gene involved in the regulation of cold dormancy. This silencing is achieved via promoter interference and histone modification, respectively [38,39]. Similarly, lncRNAs have been shown to play pivotal roles in regulating bud dormancy in pears. For example, the lncRNA *PpL-T31511* regulates pear bud dormancy by generating a microRNA (Pp-miRn182), which targets *PpPP2C*, a key gene in the ABA signaling pathway. *PpL-T31511* has been shown to induce the release from dormancy of pears via the PP2C-H2O2 pathway [40]. In this study, we successfully identified 4602 novel genes and 24,500 novel transcriptions, including 4544 lncRNAs (Figure 3D, Supplementary Tables S3, S4, and S7). This has further improved the transcriptional annotation of the pear reference genome and provided a solid data foundation for studying the role of these non-coding RNAs in the regulation of endodormancy in pears [17]. Previous studies have demonstrated that lncRNAs can *cis*-regulate the expression of neighboring genes within their genomic loci [41,42]. LncRNAs also exhibit potential as ceRNAs, sequestering miRNA pools through endogenous competition, thereby reducing miRNA-mediated transcriptional degradation of target genes, and ultimately enhancing their expression levels [43]. In our prediction results of lncRNA-mediated regulation (Supplementary Table S9), multiple novel lncRNAs were found to have the potential to cis-regulate flowering and dormancy-related genes. Among these, lncRNA ONT.1662.5 was identified as a *cis*-target of gene *ELF3* (EVM0015266), a member of the EARLY FLOWERING gene family. The lncRNAs *ONT.19912.1* and *ONT.20662.7* were identified to potentially cis-regulate *DAM1/2/3* genes. Sequence alignment further revealed that *ONT.20662.7* shares homology with the *DAM* gene family, a key regulator of bud dormancy (Figure 8, Supplementary Figure S2, and Table S9). Furthermore, a significant positive correlation was observed between the expression levels of the two aforementioned genes and those of *DAM1/2/3* genes. This suggests that these lncRNAs may play a regulatory role in the expression of *DAM1/2/3* genes during dormancy progression, although their precise mechanisms of action require further experimental validation.

## 4. Materials and Methods

### 4.1. Plant Materials

‘Mixue’ pear trees were cultivated in Qingliu county, Sanming city, Fujian, China. All the trees were spaced at 4 m×5 m intervals and received a standard horticultural practice such as irrigation, sod cultivation, and pest/disease prevention in a rainproof greenhouse facility. Based on the local temperature conditions of Qingliu County, flower bud samples were collected at four developmental stages, spanning from before the onset of chilling accumulation (defined as three consecutive days with mean temperatures below 7.2 °C) to pre-bud break (Figure 1). Sampling was conducted on 18 November 2022 (S1), 13 December 2022 (S2), 10 February 2023 (S3), and 15 March 2023 (S4), respectively. All samples were collected at midday under sunny conditions. Surface bud scales were removed to retain the internal floral bud tissues. Three biological replicates were established for each developmental stage, each comprising a composite of at least five individual buds.

### 4.2. Metabolomic Analysis of Phytohormone Profile

Fresh plant samples were immediately frozen in liquid nitrogen and pulverized using a cryogenic grinder, Osaka, Japan (30 Hz, 1 min), and the resulting powder was stored at −80 °C. A 50 mg aliquot of homogenized powder was mixed with 1 mL of methanol/water/formic acid (15:4:1, *V*/*V*/*V*) solution. Subsequently, 10 μL of an internal standard mixed solution (100 ng/mL) was added into the extract as an internal standard (IS) for quantification. After vigorous vortexing (10 min), the mixture was centrifuged at 12,000 rpm for 5 min at 4 °C, and the supernatant carefully collected. Phytohormone contents were analyzed by MetWare, Woburn, MA, USA, based on the AB Sciex QTRAP 6500 LC-MS/MS platform. Based on the self-built database and the public database of metabolite information, the test samples were examined using the multi-reaction monitoring model (MRM). Mass spectrometric data acquisition was performed using Analyst 1.6.3 software (Sciex, Framingham, MA, USA). Multiquant 3.0.3 software (Sciex, Framingham, MA, USA) was used to quantify all metabolites. Identified metabolites were annotated using the KEGG, MWDB, MassBank, KNAP-SAcK, and HMDB databases. DPMs between groups were determined based on absolute fold change thresholds (|Fold Change| ≥ 2 or ≤0.5). Clustering heatmaps and differential metabolite correlation analyses were visualized using the pheatmap package in the R language environment.

### 4.3. Full-Length Transcriptome Data Processing and Analysis

Total RNA was isolated from each sample using TRIzol reagent (Invitrogen, Carlsbad, CA, USA), followed by DNase I (Takara, Kusatsu, Japan) treatment to eliminate genomic DNA contamination. One microgram of total RNA was utilized for cDNA library construction following the cDNA-PCR Sequencing Kit protocol (SQK-LSK110 + EXP-PCB096, Oxford Nanopore Technologies, Oxford, UK). DNA purification was performed using Agencourt XP beads according to the ONT protocol. The final cDNA library was loaded onto a FLO-MIN109 flow cell for sequencing on the PromethION platform (Oxford Nanopore Technologies, Oxford, UK). Clean RNA sequencing data was deposited in the National Genomics Data Center database (https://www.cncb.ac.cn) with BioProject number ‘PRJCA032463’. The reference genome database and gene annotation files were extracted from ‘Cuiguan’ pear genome in Rosaceae database [17]. rRNA was removed after mapping to the Silva rRNA database (www.arb-silva.de). Consensus sequences were then mapped to the reference genome using minimap2 (v2.16) [44]. Mapped reads were further collapsed by the cDNA_Cupcake package with min-coverage = 85% and min-identity = 90%. Differences at the 5′ end were not considered when collapsing redundant transcripts. Count Per Million (CPM), which was calculated by the EdgeR package in the R language (v4.2.1), was used to quantify the gene expression level among the different bud samples [45]. Transcripts longer than 200 nt in length and possessing a multi-exonic architecture (≥2 exons) were initially selected as lncRNA candidates. These transcripts were then subjected to a combinatorial screening pipeline consisting of four computational methods—the Coding Potential Calculator (CPC, v0.9-r2) [24], Coding–Non-Coding Index (CNCI, v2) [22], Coding Potential Assessment Tool (CPAT, v1.2.2) [23], and Pfam 36.0 domain analysis [25]—to distinguish the protein-coding transcripts from the non-coding genes.

### 4.4. Proteome Quantification and Analytical Workflow

Protein extraction and trypsin digestion of the pear buds were performed according to previously published protocols [46,47]. Samples were ground into powder using liquid nitrogen, and mixed with lysis buffer (containing 1% TritonX-100, 10 mM dithiothreitol, 1% protease inhibitor cocktail, 50 μM PR-619, 3 μM TSA, 50 mM NAM, 2 mM EDTA, and 1% phosphatase inhibitor for phosphorylation). This was followed by three cycles of high-intensity ultrasonic treatment on ice using an ultrasonic processor (Scientz, Ningbo, China). An equal volume of Tris-saturated phenol (pH 8.0) was added and vortexed for 5 min. After centrifugation at 5000× *g* for 10 min at 4 °C, the upper phenolic phase was collected. Proteins were precipitated by adding at least four volumes of ammonium sulfate-saturated methanol, followed by incubation at −20 °C for a minimum of 6 h. After centrifugation at 5000× *g* for 10 min at 4 °C, the supernatant was discarded. The remaining pellet was washed once with ice-cold methanol followed by three washes with ice-cold acetone. The proteins were redissolved in 8 M urea, and protein concentration was determined using a BCA kit according to the manufacturer’s instructions. LC-MS/MS was conducted as previously described [48]. The tryptic peptides were dissolved in a solution of 0.1% formic acid and 2% acetonitrile in water, and subsequently loaded onto a homemade reversed-phase analytical column (25 cm length, 100 μm i.d.). The peptides were introduced into a capillary source followed by the timsTOF Pro (Bruker Daltonics, Billerica, MA, USA) mass spectrometry with a parallel accumulation serial fragmentation (PASEF) mode. The electrospray voltage applied was 1.60 kV. Precursors and fragments were detected at the TOF detector, with an MS/MS scan range from 100 to 1700 m/z. Precursors with charge states 0 to 5 were selected for fragmentation, and 10 PASEF-MS/MS scans were acquired per cycle. Dynamic exclusion was set to 30s. The resulting MS/MS data were processed using the MaxQuant search engine (v.1.6.15.0). Tandem mass spectra were searched against the SwissProt database concatenated with a reverse decoy database [49]. Trypsin/P was specified as the cleavage enzyme, allowing up to 2 missing cleavages. FDR was adjusted to below 1%.

### 4.5. Bioinformatics Methods

DEGs among the different pear buds were identified based on the pairwise comparison statistical values (FDR < 0.05, |fold change| > 2) calculated by the EdgeR (v4.0) package [45]. Time series gene expression analysis was performed using Short Time-Series Expression Miner (STEM) (v1.3.13) [50]. DEGs within 100 kilobases of the differentially expressed lncRNAs were selected as candidate *cis*-targets of lncRNAs. The LncTar software (v1.0) was used to predict candidate trans targets of lncRNAs [51]. Gene expression cluster and heatmap plotting were conducted using the pheatmap package. Promoter responsive elements were predicted using the online software PlantCARE, accessed on 10 May 2024 [52]. The prediction of transcriptional *cis*-element binding sites in the promoter region of *DAM* genes (2000 bp upstream of the transcription start site) was performed using the online software FIMO (https://meme-suite.org/meme/doc/fimo.html), accessed on 10 May 2024.The position frequency matrices (PFMs) of TFs were analyzed based on the plantTFDB database, accessed on 10 May 2024 [53]. Transcription regulatory networks were generated by combining Pearson correlation coefficients (PCC) between structural genes and TFs (PCC > 0.8) and the prediction of *cis*-element binding sites in the promoter regions of structural genes. Key genes associated with hormone metabolism and the co-expression network were visualized using the Cytoscape software (v3.7.1) [54].

## 5. Conclusions

Bud dormancy is a physiological adaptation that enables higher plants to survive cold winter conditions. In this study, we integrated hormone metabolome, proteome, and full-length transcriptome data from ‘Mixue’ pear buds across four stages of dormancy progression, creating a comprehensive multi-omics atlas to elucidate the molecular networks underlying dormancy transitions in low-CR pear cultivars. Full-length transcriptome data were used to refine the annotation of the reference genome. A total of 17,091 gene boundaries (including transcription start/termination sites) were redefined. Additionally, 14,112 5′UTRs and 14,917 3′UTRs were extended, while 21,419 alternative splicing (AS) events across 8995 genes were annotated. These improvements provide a comprehensive and high-confidence genomic resource to support functional genomics research. In the expression pattern clustering analysis, both DEPs and DEGs displayed similar developmental stage-specific clustering. Among the dormancy-specific DEGs, five members of the *DAM* gene family were identified as key regulators of bud dormancy. Correlation analysis with targeted hormone metabolomics data and promoter *cis*-element annotation suggested that ABA, GA, and SA likely regulate bud dormancy by modulating the expression levels of the *DAM* gene family. Analysis of the lncRNAs identified one *DAM* gene family homolog (*ONT.20662.7*) that may have potential *cis*-regulatory relationships with *DAM1/2/3*. However, the specific molecular mechanisms underlying these interactions require further experimental validation. By constructing a DAM-centered regulatory network integrated with hormone metabolite profiles, 35 TFs were identified as being strongly correlated with hormone levels and potentially interacting with DAM genes. Notably, a unique RVE–DAM–COL regulatory pathway was characterized, which may play a central role in regulating dormancy progression in the ‘Mixue’ low-CR pear cultivar.

## Figures and Tables

**Table 1:** The correlation analysis between DAM genes and DPMs.

Compounds	Class	DAM1	DAM2	DAM3	DAM4-1	DAM4-2
Abscisic acid	ABA	0.74	0.68	0.86	0.92	0.81
3-Indoleacetonitrile	Auxin	−0.84	−0.85	−0.71	−0.62	−0.78
Indole-3-acetic acid	Auxin	−0.75	−0.64	−0.81	−0.90	−0.85
Indole-3-acetyl-L-aspartic acid	Auxin	−0.77	−0.69	−0.70	−0.71	−0.80
Indole-3-carboxylic acid	Auxin	−0.76	−0.83	−0.79	−0.70	−0.71
L-tryptophan	Auxin	−0.64	−0.55	−0.57	−0.60	−0.68
Methylindole-3-acetate	Auxin	0.91	0.84	0.95	0.99 *	0.96
2-Methylthio-cis-zeatin riboside	CK	−0.97	−0.93	−0.95	−0.94	−0.99 *
cis-Zeatin-O-glucoside riboside	CK	0.95	0.94	0.87	0.81	0.92
Dihydrozeatin-7-glucoside	CK	0.93	0.96	0.83	0.73	0.86
trans-Zeatin-O-glucoside	CK	0.91	0.85	0.87	0.87	0.93
1-Aminocyclopropanecarboxylic acid	ETH	−0.81	−0.73	−0.78	−0.81	−0.86
Gibberellin A19	GA	−0.81	−0.84	−0.87	−0.81	−0.79
Gibberellin A20	GA	0.83	0.75	0.92	0.97	0.90
Gibberellin A24	GA	−0.86	−0.80	−0.80	−0.79	−0.88
Gibberellin A53	GA	−0.95	−0.90	−0.91	−0.90	−0.96
Gibberellin A9	GA	−1.00 **	−0.97	−0.97	−0.94	−0.99 **
Jasmonic acid	JA	0.58	0.65	0.41	0.26	0.48
Salicylic acid	SA	−0.99 **	−0.98 *	−0.95	−0.90	−0.98
Salicylic acid2-O-β-glucoside	SA	−0.50	−0.35	−0.58	−0.72	−0.63

Note: ABA, abscisic acid; CK, cytokinin; ETH, ethylene; GA, gibberellin; JA, jasmonic acid; SA, salicylic acid. * and ** indicate that the correlation coefficient is statistically significant. The former represents 0.05 < *p* < 0.01 and the latter represents *p* < 0.01.

## Data Availability

The raw sequencing data of RNA-seq was deposited in National Genomics Data Center (2024) database [55] with BioProject number “PRJCA032463”.

## Supplementary Materials

The following supporting information can be downloaded at: https://www.mdpi.com/article/10.3390/plants14142172/s1, Figure S1: The statistical histogram of the correlation coefficient between protein expression levels of proteomes and corresponding gene expression levels of reference genomes; Figure S2: The multiple sequence alignment of lncRNA *ONT.20662.7* and *DAM* gene family.; Table S1: The accumulation levels of phytohormone-targeted DPMs in buds of the ‘Mixue’ cultivar across four developmental stages; Table S2: The summary of full-length transcriptome sequencing quality in 4 different bud developmental stages of the ‘Mixue’ cultivar; Table S3: The annotation of novel genes in 4 different bud developmental stages of the ‘Mixue’ cultivar; Table S4: The information of novel transcriptions in 4 different bud developmental stages of the ‘Mixue’ cultivar; Table S5: The gene boundary information has been optimized through the utilization of full-length transcriptome data; Table S6: The alternative splicing events identified in the full-length transcriptome; Table S7: The summary of long non-coding RNA (lncRNA) identification in 4 different bud developmental stages of the ‘Mixue’ cultivar; Table S8: The summary of full-length transcriptome sequencing quality in 4 different bud developmental stages of the ‘Mixue’ cultivar; Table S9: The regulatory prediction of lncRNAs identified from full-length transcriptomes; Table S10: The expression levels of difference expression genes (DEGs) in 4 different bud developmental stages of the ‘Mixue’ cultivar; Table S11: The expression levels of proteins identified in the proteomics in 4 different bud developmental stages of the ‘Mixue’ cultivar; Table S12: The expression levels of difference expression proteins (DEPs) in 4 different bud developmental stages of the ‘Mixue’ cultivar; Table S13: The summary of response elements in promoters of the *DAM* gene family; Table S14: The information of *DAM* family genes; Table S15: The expression levels of regulatory network genes in 4 different bud developmental stages of the ‘Mixue’ cultivar; Table S16: The correlation analysis of lncRNA and DAM gene family expression levels.

## Abbreviations

The following abbreviations are used in this manuscript:

DEG	Differentially expressed gene
DEP	Differentially expressed protein
DPM	Differentially phytohormone metabolites
CR	Chilling requirement
ABA	Abscisic acid
GA	Gibberellin
SA	Salicylic acid
JA	Jasmonic acid
BR	Brassinosteroid
SL	Strigolactone
ETH	Ethylene
CK	Cytokinin
AS	Alternative splicing
CPM	Count per million
SVP	Short Vegetative Phase
TF	Transcription factor
MRM	Multi-reaction monitoring model
PASEF	Parallel accumulation serial fragmentation
LncRNA	Long non-coding RNA
PFMs	Position frequency matrices
UTR	Untranslated region
PCC	Pearson correlation coefficient
