# Supplementary material for: Integrated Multi-Omics Reveals DAM-Mediated Phytohormone Regulatory Networks Driving Bud Dormancy in ‘Mixue’ Pears
Source: Plants (Basel). 2025 Jul 14;14(14):2172. doi: 10.3390/plants14142172 (PMC12301059; doi:10.3390/plants14142172)
Supplement: Supplementary file 1 [file plants-14-02172-s001.zip › Supplementary figures.pdf]

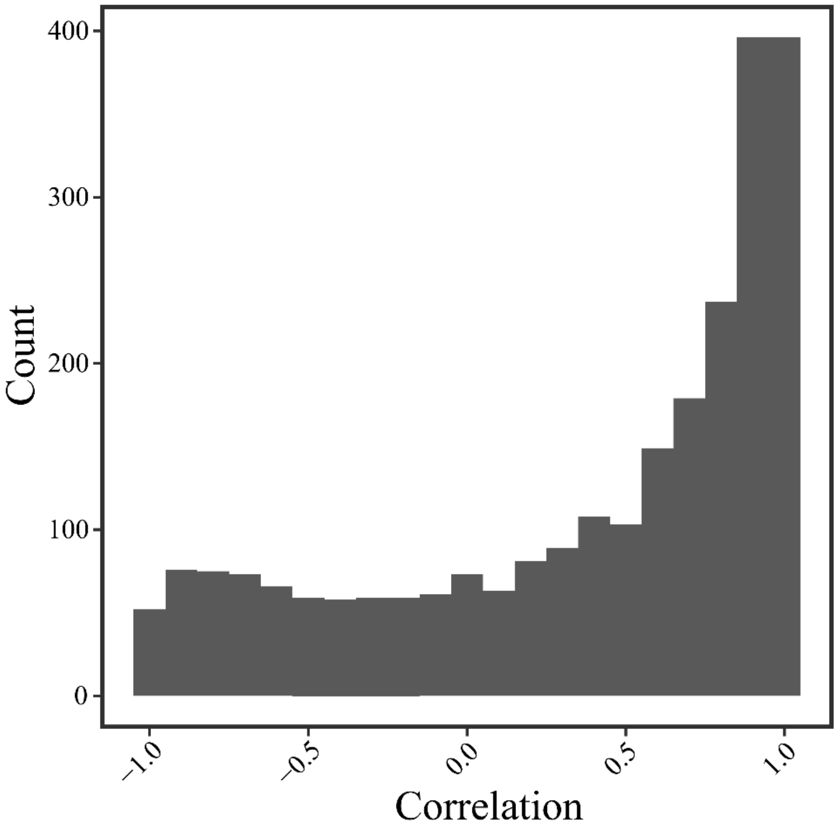

Supplementary Figure S1 The statistical histogram of the correlation coefficient between protein expression levels of proteomes and corresponding gene expression levels of reference genomes.

```
ONT.20662.7   GCCGGAGGAGATATCAGACCTGCGGCGATCGTGGAGTAGG   730
DAM1          GCTAGAGGAGATATCGAACCGGCGGCGATCATGGAGTTGG   571
DAM2          GCTGGAGGAGATATCGGACCTGCAGGGATCATGGAGTTGG   571
DAM3          GCCCGAGGAGATATCGGACCGACGGCGATCAAGGAGTTGG   599
DAM4-1        ...GGAGGAGATAGTGGACCGGA...............GG   535
DAM4-2        ...GGAGGAGATAGTGGACCGGA...............GG   535
Consensus     gc ggaggagatatcggaccggcggcgatcatggagttgg

ONT.20662.7   AAAACCTG...AATGTTGGAGAAGAAGGCGTGACATCTGA   767
DAM1          AAAACCTGAATAATGTTGGAGAAGAAGGCATGACATCTGA   611
DAM2          ACAACCTGAATAATGTTGGAGAAGAAGGCGTGACATCTGA   611
DAM3          A.AACCTGAATAATGGTGGAGAAGAAGGCGTGACATCTGA   638
DAM4-1        CCATCCTGAATAATATTGGAGAAGAAAGCGTGACATCTGA   575
DAM4-2        CCATCCTGAATAATATTGGAGAAGAAAGCGTGACATCTGA   575
Consensus     acaacctgaataatgttggagaagaaggcgtgacatctga

ONT.20662.7   ATCAGCCACAAATGTGACCACCTGCTCCAGCCGTGCT..C   805
DAM1          ATCAGCCACAAATGTCACCGCCTGCTCCAGCAGTGCTCAC   651
DAM2          ATCAGCCACAAATGTCACCACCTGCTCCAGCAGTGCTTTT   651
DAM3          ATCAGCTACAAATGTCACCATCTGCTCCAACAGTCCTCTT   678
DAM4-1        ATCAGCCACAAATGTCACCACCTTCTCCAACAGTTCTCTT   615
DAM4-2        ATCAGCCACAAATGTCACCACCTTCTCCAACAGTTCTCTT   615
Consensus     atcagccacaaatgtcaccacctgctccaacagtgctctt

ONT.20662.7   TCTCTTGAAAATGACTGCTACGACTTCTTTTTTCTCAAAC   845
DAM1          TCTCTTGAAGATGACTGTTCCGACATCTTGTCTCTCAAAC   691
DAM2          TCTCTCGAAGATGACTGCTCCGACATCTTGTCTCTCAAAC   691
DAM3          TCTCTTGACGATGACTGCTCCGACATCTTGTCTCTCAAAC   718
DAM4-1        TCCCTTGAAGATGACTGCTCCGATACCTTGTCTCTCAAAC   655
DAM4-2        TCCCTTGAAGATGACTGCTCCGATACCTTGTCTCTCAAAC   655
Consensus     tctcttgaagatgactgctccgacatcttgtctctcaaac

ONT.20662.7   TGGGGTGAGGTTCT                             859
DAM1          TGGGGCTTCCTTAG                             705
DAM2          TGGGGCTTCCTTAG                             705
DAM3          TGGGGTGA......                             726
DAM4-1        TGGGGCTTCCGTAG                             669
DAM4-2        TGGGGCTTCCGTAG                             669
Consensus     tggggcttccttag
```

Supplementary Figure S2 The multiple sequence alignment of lncRNA *ONT.20662.7* and *DAM* gene family.
